# Development of a New Swine Model Resembling Human Empty Nose Syndrome

**DOI:** 10.3390/medicina60101559

**Published:** 2024-09-24

**Authors:** Dan Bi Park, David W. Jang, Do Hyun Kim, Sung Won Kim

**Affiliations:** 1 Postech-Catholic Biomedical Engineering Institute, College of Medicine, The Catholic University of Korea, Seoul 06591, Republic of Korea; bdb613@naver.com; 2 Department of Head and Neck Surgery & Communication Sciences, Duke University School of Medicine, Durham, NC 27710, USA; david.jang@duke.edu; 3 Department of Otolaryngology-Head and Neck Surgery, Seoul Saint Mary’s Hospital, College of Medicine, The Catholic University of Korea, Seoul 06591, Republic of Korea

**Keywords:** empty nose syndrome, atrophic rhinitis, animal model, swine, turbinoplasty

## Abstract

*Background and Objectives:* Empty nose syndrome (ENS) is a debilitating condition that often results from traumatic or iatrogenic causes, such as nasal surgery. There are various conservative and surgical treatments for ENS, but their effectiveness remains uncertain. Therefore, the development of animal models that accurately mimic human ENS is essential for advancing effective treatment strategies. *Materials and Methods:* To investigate ENS development, turbinoplasty was performed in the nasal cavity of swine, entailing partial removal of the ventral turbinate using turbinectomy scissors followed by electrocauterization. After 56 days, samples were obtained for histological and morphological analyses. *Results:* A significant reduction in the volume of the ventral turbinate in the ENS model led to an expansion of the nasal cavity. Histological analysis revealed mucosal epithelial changes similar to those observed in ENS patients, including squamous cell metaplasia, goblet cell metaplasia, submucosal fibrosis, and glandular atrophy. Biomarkers related to these histopathological features were identified, and signals potentially contributing to squamous cell metaplasia were elucidated. *Conclusions:* The swine ENS model is anticipated to be instrumental in unraveling the pathogenesis of ENS and may also be useful for evaluating the effectiveness of various treatments for ENS.

## 1. Introduction

Empty nose syndrome (ENS) is an iatrogenic condition that develops subsequent to nasal surgery and particularly affects the turbinates. In 1994, Kern and Stenkvist coined this term to describe the marked reduction in intranasal tissue surrounding the inferior and middle turbinates [1]. ENS occurs in up to 16% of patients undergoing turbinate surgery, and patients can experience ENS months to years after the surgery [2,3]. 

Typically, ENS patients exhibit nasal-cavity symptoms such as excessive nasal crusting, mucosal dryness, paradoxical nasal obstruction, nosebleeds, and a mucus-filled runny nose. Less commonly, symptoms such as headache, facial pain, anosmia, difficulty breathing, disrupted sleep, and emotional disturbances including depression, anger, anxiety, fatigue, irritability, and frustration can cause serious psychological problems, even suicide [4,5]. Medical treatment for ENS is conservative, including hydrating the nasal passages with nasal saline spray or oil-based lubricants, prescribing anti-depressants, increasing fluid intake, and intermittently closing the nostrils to restore humidity [6,7]. This treatment is somewhat effective, but it is not curative. Consequently, the number of ENS patients is growing.

Recent studies have indicated that surgical interventions and mucosal regenerative strategies can lead to sustained alleviation of ENS symptoms. However, there is generally marginal clinical improvement in up to 21% of patients [8]. To develop effective ENS treatments, animal models of human ENS are needed. However, no animal model of ENS following turbinate surgery, which is the most common cause, has been reported. Therefore, in this study, we attempted to create an animal model of ENS and examined the underlying mechanisms of onset that can be targeted for the development of therapeutic strategies.

## 2. Materials and Methods

### 2.1. Ethics Approval

All animals involved in this experiment were cared for following ethical standards. All procedures were conducted in compliance with applicable ethical standards and received approval from the Institutional Animal Care and Use Committee (IACUC) of CRONEX (CRONEX-IACUC Approval No. 202309-002). They were maintained under controlled conditions in a standard laboratory setting in accordance with IACUC guidelines. Only animals that passed thorough screening for parasites and microbial infections, as per IACUC quarantine guidelines, were utilized in this study.

### 2.2. Surgical Procedure

**Figure 1:**
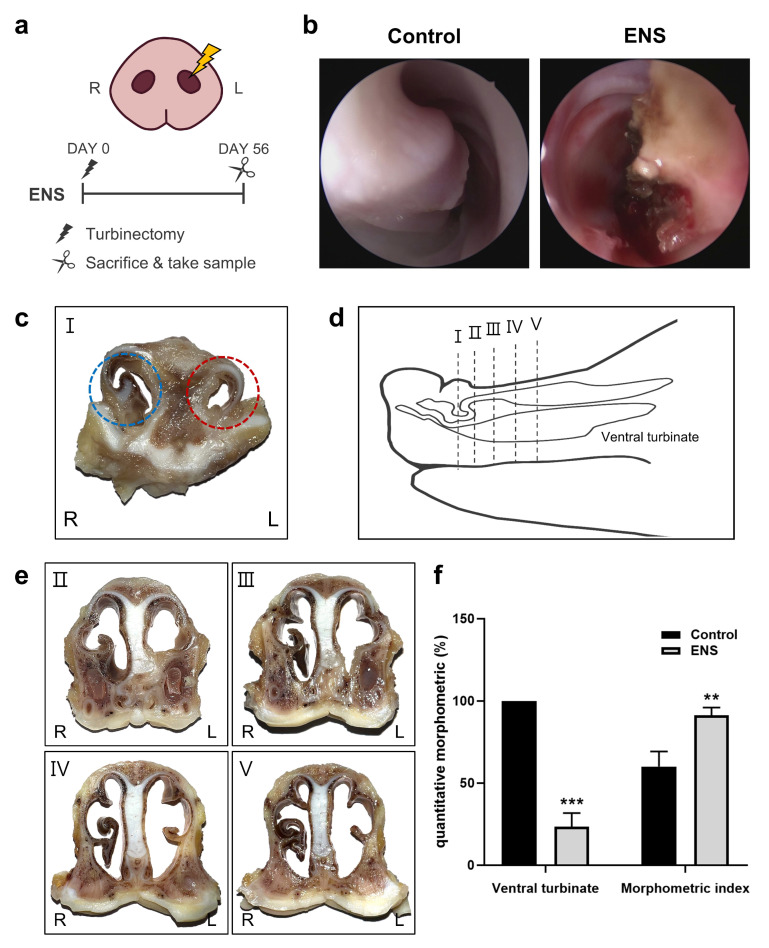
Morphology of the nasal cavity following turbinectomy on the swine snout. (**a**) Overview of the surgical procedure. (**b**) Endoscopic image of the swine ventral turbinate. (**c**) Cross-sectional view of the ventral turbinate: control (blue) and ENS after turbinoplasty (red). (**d**) Lateral view and cross-section of the swine snout. (**e**) Nasal cavity cross-sections at various locations. (**f**) Ventral turbinate volume and morphometric index; data are means ± standard deviation (SD) (** *p* < 0.01, *** *p* < 0.001).

The experimental procedure involved the use of a single female conventional swine weighing 40–50 kg. Because this trial was conducted as a pilot study to determine whether ENS could be induced via turbinoplasty, no sample-size calculation was performed. Turbinoplasty was performed on the left nasal cavity of the swine under isoflurane inhalational anesthesia, with the right nasal cavity serving as a control. The nasal cavity was examined using a 4 mm endoscope, and the ventral turbinate (equivalent to the human inferior turbinate) was partially removed using turbinectomy scissors. Hemostasis was achieved by cauterization of the mucosa using bayonet-type forceps and a ValleyLab Force FX™ Electrosurgical Generator (Medtronic, Minneapolis, MN, USA) at 15 W for 3–5 s. This procedure facilitated turbinate electrocauterization and bone repositioning. After 56 days, the animals were euthanized by an overdose of sodium pentobarbital injection and samples were collected to evaluate the procedure outcomes (Figure 1a). All surgical interventions were performed by a rhinology specialist.

### 2.3. Hematological Analysis

Blood samples were collected from the swine before turbinectomy was performed and at euthanasia and were transferred to heparin-coated tubes to separate serum and blood cells. Serum hematological parameters were analyzed using a Hitachi 7180 clinical analyzer (Hitachi clinical analyzer 7180; Hitachi, Tokyo, Japan), following the manufacturer’s protocol. The obtained hematological values were compared with established normal ranges to assess abnormalities [9,10].

### 2.4. Scanning Electron Microscopy (SEM)

Turbinate tissue was fixed overnight using a 2.5% glutaraldehyde buffered solution and then rinsed twice with 0.1 M phosphate buffer and post-fixed with 2% osmium tetroxide for 2 h. Following fixation, the samples were dehydrated using a series of graded ethanol solutions and allowed to dry. Then, they were sputter-coated with platinum using the SMC12R-Plus system (Semian, Daejeon, Republic of Korea). Finally, the samples were examined by SEM (Regulus 8220; Hitachi).

### 2.5. Tissue Processing

Following euthanasia, samples were collected and fixed in 10% formalin overnight and then washed with phosphate-buffered saline (PBS). Then, the samples were decalcified in 14% ethylenediaminetetraacetic acid solution (pH 7.8) for 2 months before they were embedded in paraffin.

### 2.6. Histological Analysis

The specimens were sliced into 4.0 μm thick sections, deparaffinized with xylene, and gradually rehydrated using a sequence of ethanol solutions. After deparaffinization, various sections were stained with hematoxylin and eosin (H and E; Daejung, Republic of Korea), Masson’s trichrome (MT) stain kit (Abcam, Cambridge, UK), and Alcian blue (Abcam), following the manufacturers’ guidelines.

### 2.7. Immunohistochemistry (IHC) and Immunofluorescence (IF) Analysis

Each section was subjected to heat-induced epitope retrieval in 0.01 M citrate buffer (Sigma-Aldrich, St. Louis, MO, USA), and then endogenous peroxidase activity was neutralized using 3% H_2_O_2_. Subsequently, the sections were blocked with 5% normal goat serum (Vector Laboratories, Newark, CA, USA) for 1 h. For IHC analysis, primary antibodies (anti-KRT5, anti-ΔNp63, and anti-MUC5AC from Abcam; anti-β-catenin and anti-SOX2 from Santa Cruz Biotechnology, Dallas, TX, USA) were applied overnight. After washing with PBS, the sections were treated with a mixture of anti-rabbit and anti-mouse horseradish peroxidase polymer (GBI Labs, Rockville, MD, USA) for 30 min. Diaminobenzidine staining (GBI Labs) was then performed to produce a dark brown color and was followed by counterstaining with hematoxylin.

For IF analysis, sections were incubated overnight with primary anti-ΔNp63 and anti-KRT13 (Santa Cruz Biotechnology) antibodies. After washing, the sections were treated with secondary anti-mouse IgG Alexa Fluor 488 and anti-rabbit IgG Alexa Fluor 647 antibodies (1:1000) from Invitrogen (Waltham, MA, USA), for 2 h. Subsequently, 4′,6-diamidino-2-phenylindole counterstaining (Invitrogen) was performed. The slides were visualized using a confocal laser microscope (LSM 800; Carl Zeiss, Oberkochen, Germany).

### 2.8. Quantitative and Statistical Analysis

Stained sections were randomly captured from three different fields for analysis under magnifications of 50× for Alcian blue, 200× for H and E and MT staining, and 400× for IHC. To quantify IHC results, we expressed the percentage of positive cells by calculating the proportion of cells with nuclear staining. Specifically, the number of positively stained nuclei was divided by the total number of nuclei in the examined field, and the result was expressed as a percentage to represent the extent of marker expression. Quantitative analysis of pixel values corresponding to the features of interest in each selected area was performed using ImageJ software (64-bit, Java 8 version). Statistical analyses, including unpaired *t*-tests to evaluate differences between groups, were conducted using Prism 8.0 software (GraphPad Software, San Diego, CA, USA). In the calculation of the morphometric index, the sample size was quantified using the sections corresponding to positions IV and V in Figure 1d. Using the measured sample size, the morphometric index was calculated as follows [11,12]:

$$\frac{Free\;space\;in\;nasal\;cavity\;}{Free\;space\;in\;nasal\;cavity\;+\;Ventral\;turbinate}\times 100(\%)$$

## 3. Results

### 3.1. Morphological Changes in the Nasal Cavity in the ENS Model

Following turbinoplasty, immediate endoscopic imaging revealed visible tissue damage (Figure 1b). Cross-sectional views of the ventral turbinate taken 56 days post-turbinoplasty showed structural changes suggestive of ENS (Figure 1c). However, even after turbinectomy and observation of the animals for 56 days, the blood biochemistry values remained within the normal range (Table 1). An examination of tissue sections revealed variation in the cross-sectional shape based on the location within the nasal cavity.

In the ENS model, increased fibrosis resulting from the defect damage narrowed the nasal cavity anteriorly, and the nasal cavity was widened beyond the defect, as observed in the tissue sections (Figure 1d,e). The ventral turbinate volume decreased significantly to 23.44%± 8.29% in the ENS model relative to the control. Furthermore, the morphometric index was 91.30% ± 4.73% in the ENS group compared to 60.02% ± 9.20% in the control, indicating significant expansion of the nasal cavity (Figure 1f).

### 3.2. Histological Changes in the Mucosal Epithelium in the ENS Model

**Figure 2:**
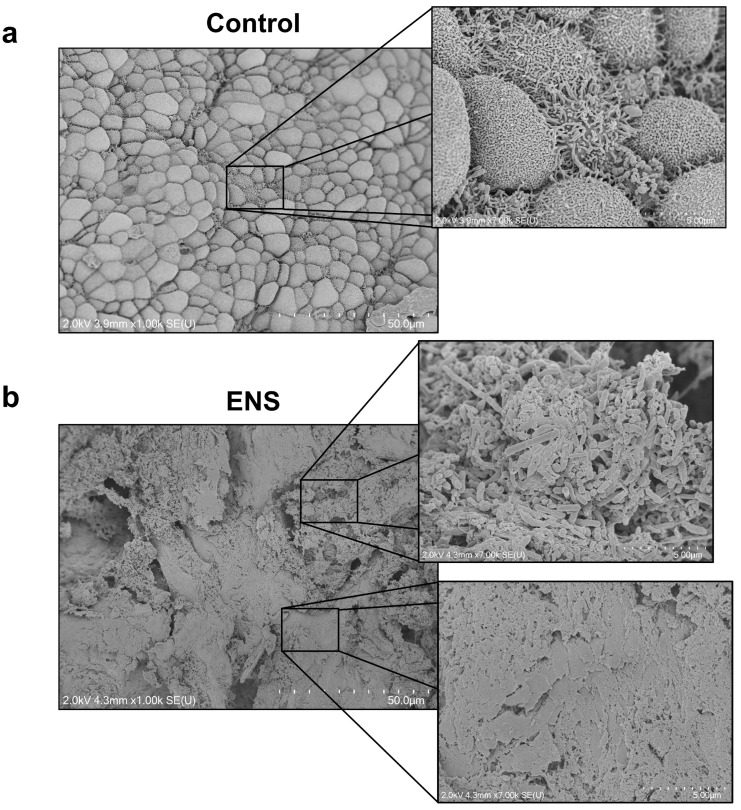
Surface changes in the ventral turbinate in the ENS model. (**a**,**b**) Surface images of the ventral turbinate captured by scanning electron microscopy at magnifications of 1000× and 7000×.

**Figure 3:**
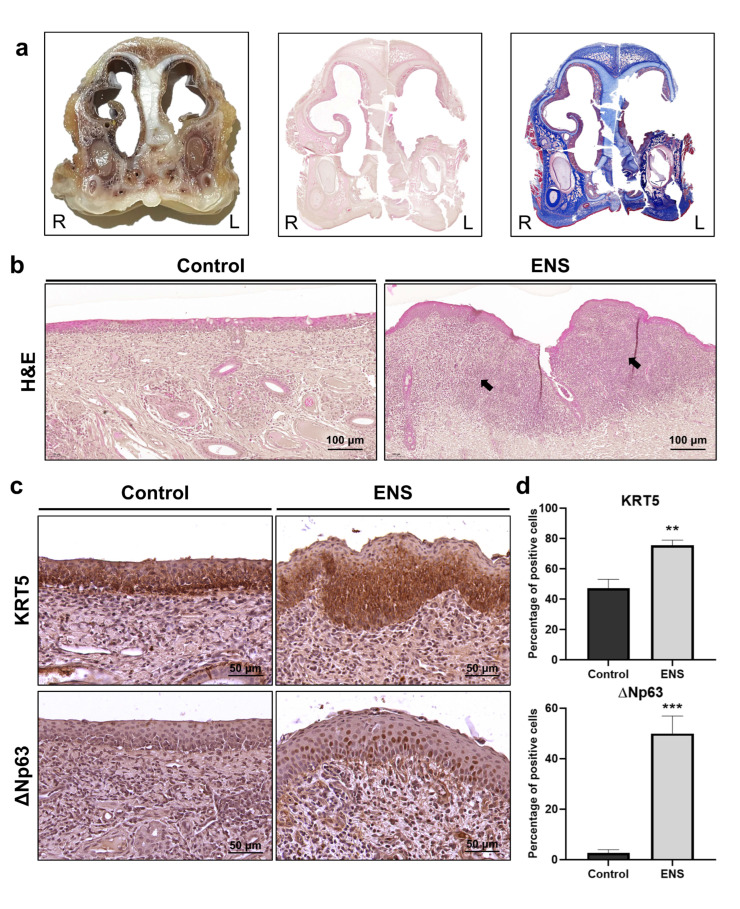
Histological alterations in the epithelium in the ENS model. (**a**) Cross-sectional images of tissue samples with H and E and MT staining. (**b**) Tissues were examined at 200× magnification following H and E staining to compare histological features between the control and ENS groups. Arrow indicates squamous cell metaplasia. (**c**) IHC staining for KRT5 and ΔNp63, examined at 400× magnification. (**d**) IHC images were compared quantitatively; data are means ± SD (** *p* < 0.01, *** *p* < 0.001).

**Figure 4:**
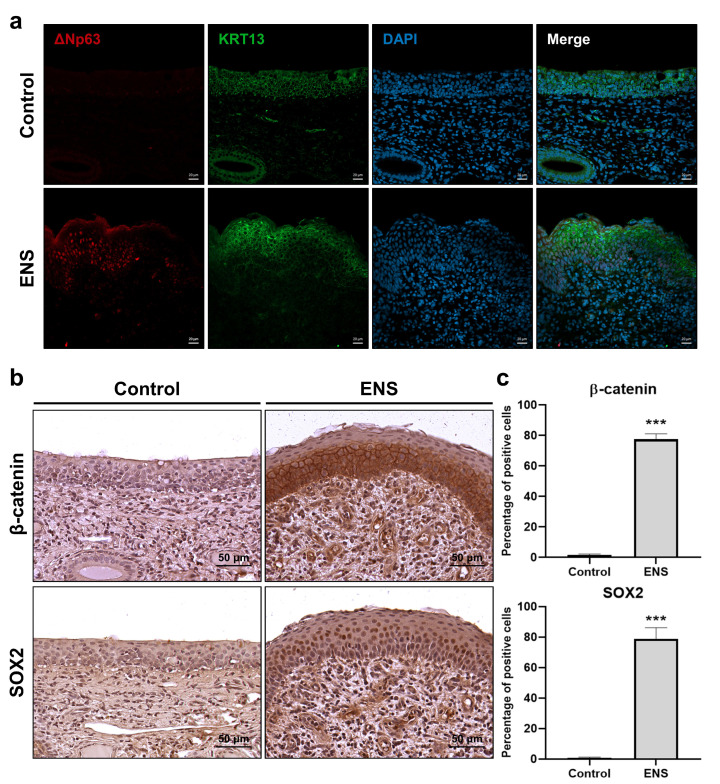
The difference in protein expression in ENS squamous epithelium. (**a**) Comparison of ΔNp63 and KRT13 co-expression between the two groups using IF staining. (**b**) IHC images of β-catenin and SOX2. (**c**) Percentages of stained cells in the epithelium; data are means ± SD (*** *p* < 0.001).

To evaluate histological changes in the ENS model, ciliary coverage was examined. Observations revealed the absence of cilia and the presence of bacilli (Figure 2a,b). H and E staining of cross-sectioned tissues showed squamous metaplasia in the ENS model (Figure 3a), in which columnar epithelium transitioned into stratified squamous epithelium (Figure 3b). Additionally, overexpression of the basal-cell markers KRT5 and ΔNp63 suggested a shift in cell composition, with levels increasing from 47.22% ± 5.9% to 75.57% ± 3.40% and from 2.66% ± 1.33% to 49.92% ± 7.0%, respectively. Co-expression of ΔNp63 and KRT13 confirmed the presence of squamous cells in the ENS model (Figure 4a). The expression levels of β-catenin and SOX2 were significantly elevated, from 1.77% ± 0.54% to 77.51% ± 3.64% and from 0.82% ± 0.45% to 78.98% ± 7.19%, respectively, suggesting their involvement in driving squamous metaplasia in the ENS model (Figure 4b,c).

**Figure 5:**
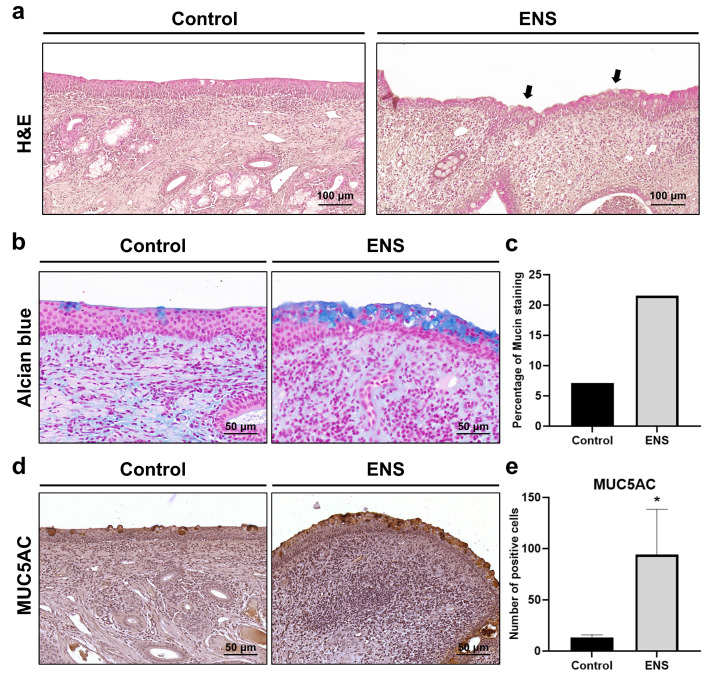
Goblet cell metaplasia was observed in the ENS epithelium. (**a**) Goblet cell metaplasia (arrows) was observed at 200× magnification following H and E staining. (**b**,**c**) Mucin were visualized following Alcian blue staining and plotted as percentages. (**d**,**e**) Goblet cell metaplasia was visualized using IHC with MUC5AC at 400× magnification and plotted as numbers of stained cells; data are means ± SD (* *p* < 0.05).

Images of H and E-stained tissues showed an increase in the number of cells with goblet cell morphology in the pseudostratified epithelium (Figure 5a). Subsequent Alcian blue staining to assess mucin expression revealed an increase from 7.12% to 21.51% (Figure 5b,c). Moreover, there was a significant increase in number of MUC5AC-positive cells in the goblet metaplasia area, from 13.33 ± 2.52 to 94.0 ± 44.4, indicating a marked increase in MUC5AC expression (Figure 5d,e).

### 3.3. Histological Modifications in Submucosal Structure in the ENS Model

**Figure 6:**
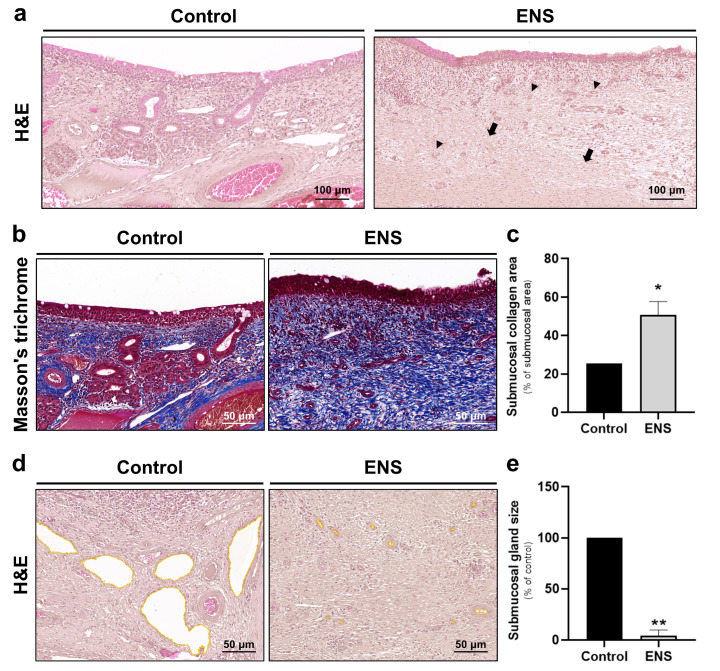
Histological changes were observed in ENS submucosa. (**a**) Histological changes in the submucosa were observed at 200× magnification following H and E staining. Arrows and arrowheads indicate submucosal fibrosis and glandular atrophy, respectively. (**b**,**c**) Collagen distributions in the submucosa were visualized using MT staining and plotted as means ± SD. (**d**,**e**) Submucosal glands (yellow lines) were visualized at 200× magnification following H and E staining, and their sizes were plotted as means ± SD (* *p* < 0.05, ** *p* < 0.01).

Submucosal fibrosis and glandular atrophy were confirmed through H and E staining (Figure 6a). Further assessment of fibrosis using MT staining showed an increase in blue-stained collagen within the submucosa (Figure 6b). The collagen-stained area as a percentage of the submucosal area increased two-fold, from 25.46% in the control to 50.89% ± 6.83% in the ENS model (Figure 6c). Additionally, H and E staining revealed a marked decrease in the size of the submucosal glands (Figure 6d) from 100.0% in the control to 4.38% ± 5.51% in the ENS model (Figure 6e).

## 4. Discussion

Surgical removal of the nasal turbinate can disrupt nasal function, potentially triggering the onset of ENS, which typically occurs after inferior turbinate resection, with a lower chance of occurrence following middle turbinate resection [13,14]. Therefore, it is necessary to identify animals in which ENS can be induced through turbinoplasty while the inferior turbinate is visualized with an endoscope. We hypothesized that animals in which atrophic rhinitis (AR) can be induced could be suitable for the induction of ENS. Atrophic rhinitis models have been developed using swine and rats, with the condition induced by toxins from *Pasteurella multocida or Bordetella bronchiseptica* [15,16]. These models differ from the model developed in the present study because they involve primary AR induced using bacterial strains and not secondary AR caused by nasal surgery. However, turbinectomy/turbinoplasty can be performed in swine using the same procedure as in humans, so we attempted to create a swine ENS model and observed whether it could replicate key characteristics seen in human patients with ENS.

In our swine model, following turbinoplasty, we observed a reduction in ventral turbinate volume. This is reminiscent of the AR induced in swine models, where atrophy of the nasal turbinates leads to an expansion of the nasal cavity’s free space. As a result, the morphometric index increased, reflecting the degree of turbinate atrophy and associated structural changes [17,18]. In animals infected with bacteria such as *Pasteurella multocida* or *Bordetella bronchiseptica*, hematological parameters such as CRP, BUN, AST and ALT showed increases [19,20,21]. However, in our study, these parameters did not show a significant increase. Instead, we observed morphological changes in the nasal cavity. This suggests that the observed changes in our model may not be attributable to bacterial infection, indicating the possibility that other factors might be responsible for these structural alterations.

In humans, these histological changes include squamous and goblet cell metaplasia in the mucosal epithelium and fibrosis and gland atrophy in the submucosa. Furthermore, keratinization is observed in some areas where squamous cell metaplasia has occurred [22,23]. In our swine ENS model, the normal respiratory columnar epithelium was transformed into stratified squamous epithelium and keratinization was observed in some areas, along with squamous cell metaplasia. This indicates that our model has the potential to effectively replicate these histological changes.

The TP63, KRT5, KRT13, and KRT15 genes are canonical markers of squamous epithelial cells, although TP63 and KRT5 are also widely recognized as canonical basal-cell markers in the airway epithelium, imposing limitations when they are used alone to identify squamous epithelial cells [24]. In a study using single-cell sequencing analysis to categorize airway basal cells into clusters, the basal cell markers TP63 and KRT5 were expressed throughout the clusters, whereas KRT13 was specifically expressed in the basal−squamous cluster [25]. The TP63 gene has two main isoforms: one retaining (TA) and the other lacking (ΔN) the transactivation domain [26]. ΔNp63 plays a role in epithelial morphogenesis, where its expression and activity are pivotal in transcriptional and post-transcriptional processes during squamous differentiation. It is predominantly expressed in stratified squamous epithelium, where it is particularly localized to the basal cell layer [27,28,29]. In the ENS model, we observed co-expression of KRT5 and KRT13, as well as overexpression of ΔNp63. This suggests that our ENS model not only replicates the histological characteristics of squamous cell metaplasia but could also have the potential to mimic its associated biomarkers.

The Wnt/β–catenin signaling pathway regulates squamous differentiation in airway basal cells [30,31]. There is a reciprocal relationship between levels of expression of the ΔNp63 gene and the Wnt/β–catenin signaling pathway. ΔNp63 can upregulate the expression of Frizzled, a Wnt ligand receptor, and the Wnt/β–catenin signaling pathway acts as an upstream regulator of ΔNp63 [32,33]. The SOX2 gene plays a crucial role in both the development and the maintenance of squamous epithelium, interacting with ΔNp63 [34]. In our ENS model, we observed significant increases in β-catenin and SOX2 expression. These two signals have co-relationships with ΔNp63. However, further research is needed to determine which gene is activated first and then influences the other during the pathogenesis of ENS.

The ENS model exhibited a significant increase in mucin expression, particularly in goblet cells. This finding aligns with the histological shift from ciliated cells to goblet cells, along with the confirmed MUC5AC expression in areas with goblet metaplasia. Interestingly, research has suggested that 35% of patients with ENS experience squamous and goblet cell metaplasia simultaneously [12,35]. 

Submucosal fibrosis was observed through the visualization of collagen fibers associated with the high collagen concentrations in fibrotic cells. The collagen-fiber area was doubled in the ENS model, although the collagen area was also high in the control. The swine vomeronasal organ consists of connective tissue containing collagen and elastin, resulting in high expression of collagen [36]. Moreover, a significant decrease in the size of the submucosal gland was evident, likely due to the progression of submucosal fibrosis, which may have further affected atrophic changes of the nasal mucosa. The resulting glandular atrophy leads to dryness in the nasal cavity and the formation of dried crusts. Moreover, the absence of cilia impedes the clearance of secretions, further facilitating crust formation [37]. 

We developed an ENS model by simulating the turbinoplasty performed in humans. Since the surgical techniques used in human procedures require experimentation on large animals, swine were selected for this study. However, in a difference from the human nasal structure, swine possess a protruding snout, which results in anatomical differences [38,39,40,41]. The effect of these anatomical discrepancies on the development of the model remains unclear. Furthermore, one of the tools for diagnosis of ENS in humans, the Empty Nose Syndrome 6-Item Questionnaire, assesses subjective symptoms such as dryness, nasal crusting, nasal burning, lack of air sensation, suffocation, and the sensation of the nose being too open [42,43]. These patient-reported outcomes are challenging to evaluate in swine. Therefore, there is a need to develop alternative diagnostic methods that can address these limitations in animal models.

## 5. Conclusions

As we noted, turbinoplasty can be performed in the swine model in a way similar to how it is performed in humans. This model may be helpful for evaluating the various surgical or mucosal regenerative approaches being developed to treat ENS. Additionally, our swine model expressed markers that are also found in ENS patients, which will provide further information on the appropriateness of the model and treatment effectiveness.

## Figures and Tables

**Table 1:** Hematological parameters.

Variables	Unit	Criterion Range	0 Day	56 Days
CRP	μg/mL	1.5~25.0	2	3
BUN	mg/dL	5.59~18.87	14	6
AST	IU/L	16.4~504	53	29
ALT	IU/L	13.2~53.2	50	51

## Data Availability

The data analyzed in this study are available from the corresponding author upon reasonable request.

## Abbreviations

Empty nose syndrome; ENS, Atrophic rhinitis; AR, Institutional Animal Care and Use Committee; IACUC, Scanning Electron Microscopy; SEM, Phosphate-buffered saline; PBS, Hematoxylin and Eosin; H & E, Masson’s trichrome; MT, Immunohistochemistry; IHC, Immunofluorescence; IF, immunofluorescence.
